# Novel Chemically-modified DNAzyme targeting Integrin alpha-4 RNA transcript as a potential molecule to reduce inflammation in multiple sclerosis

**DOI:** 10.1038/s41598-017-01559-w

**Published:** 2017-05-09

**Authors:** Madhuri Chakravarthy, May T. Aung-Htut, Bao T. Le, Rakesh N. Veedu

**Affiliations:** 1 Centre for Comparative Genomics, Discovery Way, Murdoch University, Perth, WA 6150 Australia; 2 Perron Institute for Neurological and Translational Science, Nedlands, WA 6009 Australia

## Abstract

Integrin alpha-4 (ITGA4) is a validated therapeutic target for multiple sclerosis (MS) and Natalizumab, an antibody targeting ITGA4 is currently approved for treating MS. However, there are severe side effects related to this therapy. In this study, we report the development of a novel DNAzyme that can efficiently cleave the *ITGA4* transcript. We designed a range of DNAzyme candidates across various exons of *ITGA4*. RNV143, a 30mer arm-loop-arm type DNAzyme efficiently cleaved 84% of the *ITGA4* mRNA in human primary fibroblasts. RNV143 was then systematically modified by increasing the arm lengths on both sides of the DNAzymes by one, two and three nucleotides each, and incorporating chemical modifications such as inverted-dT, phosphorothioate backbone and LNA-nucleotides. Increasing the arm length of DNAzyme RNV143 did not improve the efficiency however, an inverted-dT modification provided the most resistance to 3′ → 5′ exonuclease compared to other modifications tested. Our results show that RNV143A could be a potential therapeutic nucleic acid drug molecule towards the treatment for MS.

## Introduction

**Figure 1:**
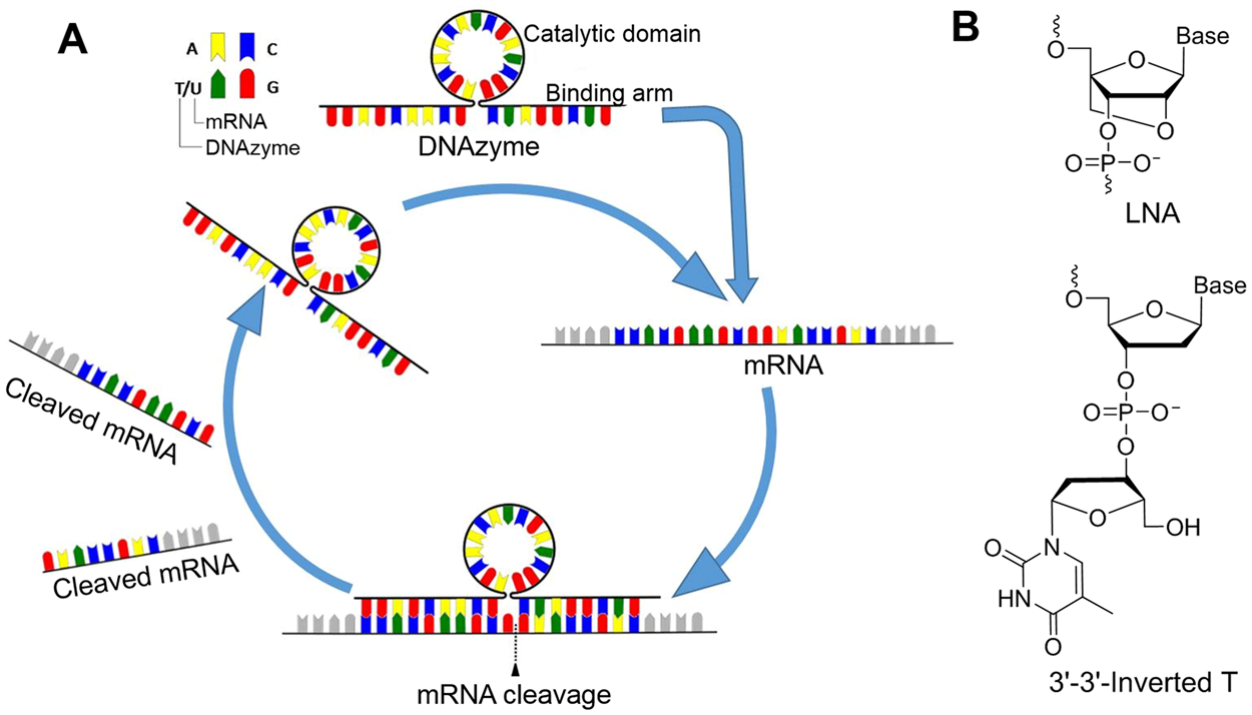
(**A**) Schematic illustration of DNAzyme-based mRNA cleavage, and (**B**) structural representations of LNA and inverted thymidine monomers.

Nucleic acid-based therapeutic technologies have gained significant interest for developing therapies targeting RNA molecules^1^. So far, four oligonucleotide drugs have been approved by the United States Food and Drug Administration for clinical use^2–5^. Vitravene, Kynamro and Eteplirsen are antisense oligonucleotides approved for the treatment of cytomegaloviral retinitis, familial hypercholestrolemia and Duchenne muscular dystrophy respectively, while a nucleic acid aptamer candidate Macugen was approved for age-related macular degeneration^2–5^. DNAzymes are another unique class of synthetic catalytic oligonucleotides that can play various roles including gene silencing and as biosensing molecules for diagnostic applications^6^. DNAzymes anneal to complementary RNA substrates through Watson-Crick base pairing rules and cleaves the phosphodiester bond mainly at purine-pyrimidine or less frequently at purine-purine junction in the presence of divalent metal ions (e.g. Mg^2+^, Ca^2+^, Pb^2+^)^7–9^. DNAzymes possess enzyme-like characteristic and the catalytic activity is dependent on divalent metal ions as cofactors^7^. The simple structure of DNAzyme includes two binding arms for specific target mRNA binding, and a catalytic core in between the binding arms to catalyse the cleavage of the target mRNA (Fig. 1A). One big advantage of DNAzymes over RNase-H dependent antisense oligonucleotides is that it can recognize and cleave the target mRNA without RNase-H recruitment and the process is reiterative (Fig. 1A).

Inflammation in the central nervous system is one of the major problems of chronic neurodegenerative disease such as multiple sclerosis (MS)^10^. ITGA4 is a validated therapeutic target for MS since a humanized monoclonal antibody, natalizumab, against ITGA4 is successful in delaying the disease progression^11^. The drug interferes with the interaction between ITGA4 and vascular cell adhesion molecule-1 and prevents immune cells from crossing the blood brain barrier and attacking the central nervous system^11^. Patients treated with natalizumab over several years have reduced or are free of annual relapses, however, there are significant limitations associated with natalizumab such as an increased risk of progressive multifocal leukoencephalopathy and development of side effects including serum sickness, generation of anti-drug antibodies and other adverse effects as discussed in recent reviews^12, 13^. Herein, we report the development of novel DNAzymes that can efficiently cleave *ITGA4* mRNA transcript as potential therapeutics to reduce ITGA4 activity in MS.

## Results

### Design and screening of first-generation DNAzymes targeting *ITGA4* mRNA

**Figure 2:**
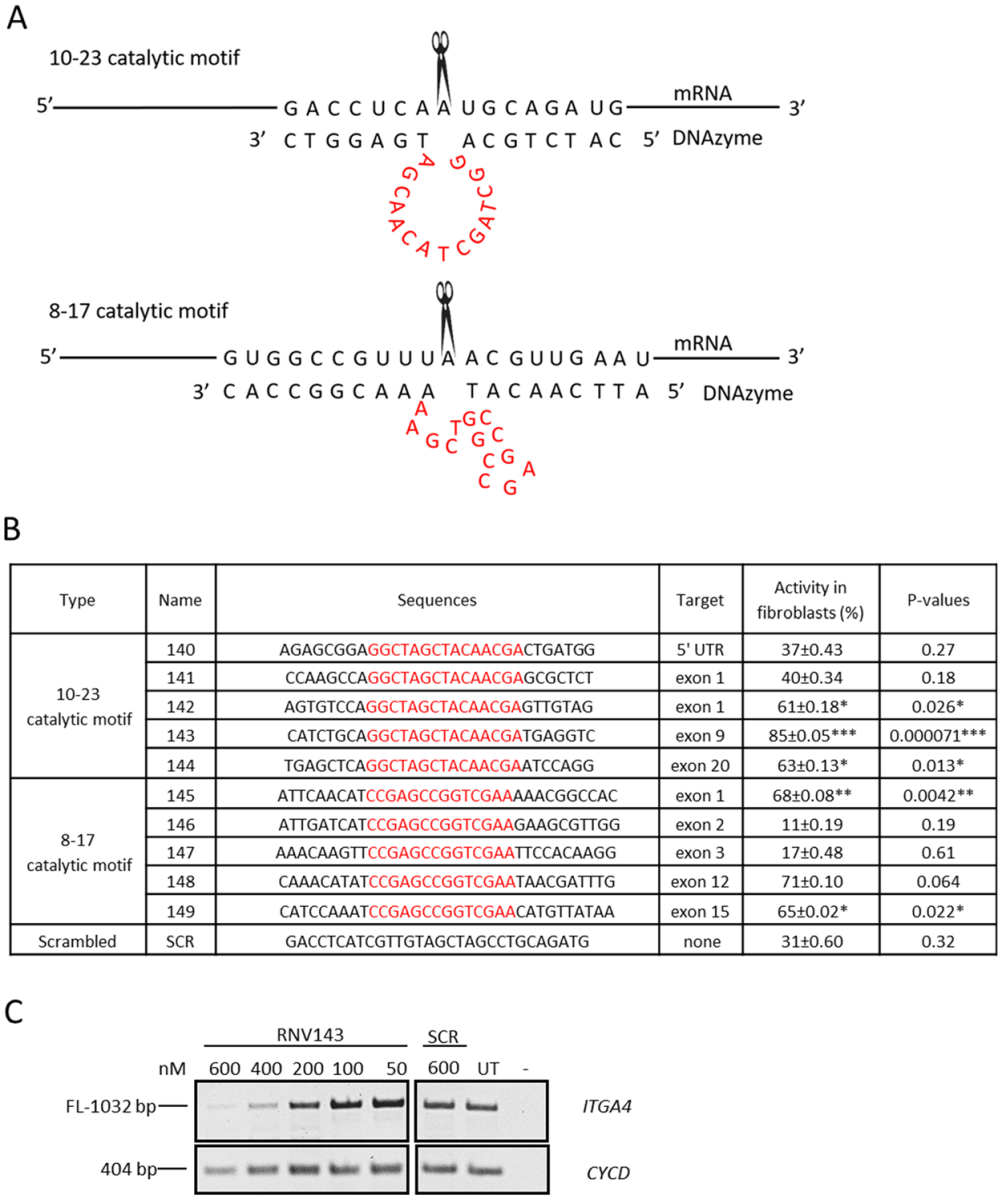
(**A**) Schematic illustration of the 10–23 and 8–17 catalytic motifs of DNAzymes. (**B**) Table for the activities of first-generation DNAzymes targeting *ITGA4* mRNA that is directly correlated with the percentages of *ITGA4* mRNA knockdown (See Materials and Methods for detailed procedures); The catalytic motifs are shown in red, the arm regions are in black, and the sequences are from 5′ → 3′. p-values were calculated for the activity in fibroblasts which was normalised to the UT using student t-test and *Indicates p-value < 0.05, **Indicates p-value < 0.005 and ***Indicates p-value < 0.0005. p-values have been rounded to 2 s.f. (**C**) Representative RT-PCR products of the *ITGA4* and *CycD* transcripts from normal human primary fibroblasts after treatment with DNAzyme at different concentrations. The RT-PCR products after treatment with RNV143 are shown here. FL, full-length; UT, untreated; SCR, scrambled sequence; *CYCD* was used as a loading control. The gel images were cropped to highlight the *ITGA4* specific products and the corresponding house-keeping gene control *CYCD*. The original images are shown in Figure S2 (Supplementary Information).

Two groups of DNAzymes, one with 10–23 catayltic motif (hammerhead) and the other with 8–17 catalytic motif (arm-loop) (Fig. 2A) were designed to target various exons of the *ITGA4* transcript. The sequences of the catalytic regions were pre-fixed according to previous reports^14, 15^ and the arm regions were designed to be specific and complementary to the *ITGA4* mRNA sequences (Fig. 2B). The catalytic activities of these DNAzymes against *ITGA4* mRNA were screened in human primary fibroblasts by transfecting DNAzymes at different concentrations (600 nM, 400 nM, 200 nM, 100 nM and 50 nM) for 24 h. RNA was extracted from the cell lysate and the integrity of *ITGA4* transcript was assessed by performing RT-PCR. Scrambled sequence (SCR) was used as a negative control to account for the non-specific effects.

Dose-dependent reduction of the full-length *ITGA4* transcript was observed for all DNAzymes treated samples. The best *ITGA4* transcript knockdown was observed at 600 nM and therefore, the efficiency of each DNAzyme was calculated as a percentage of *ITGA4* transcript knockdown at 600 nM after normalising to the house keeping gene transcript cyclin D (*CYCD*) and described as the activity in fibroblasts (Fig. 2B). DNAzyme candidate RNV143 targeting the exon 9 of the *ITGA4* mRNA showed highest efficacy with 84% knockdown of *ITGA4* (gel shown in Fig. 2C) followed by RNV148 (71% although this was not statistically significant) and RNV145 (68%) (Fig. 2B).

### Design and screening of second-generation DNAzymes targeting *ITGA4* mRNA

**Figure 3:**
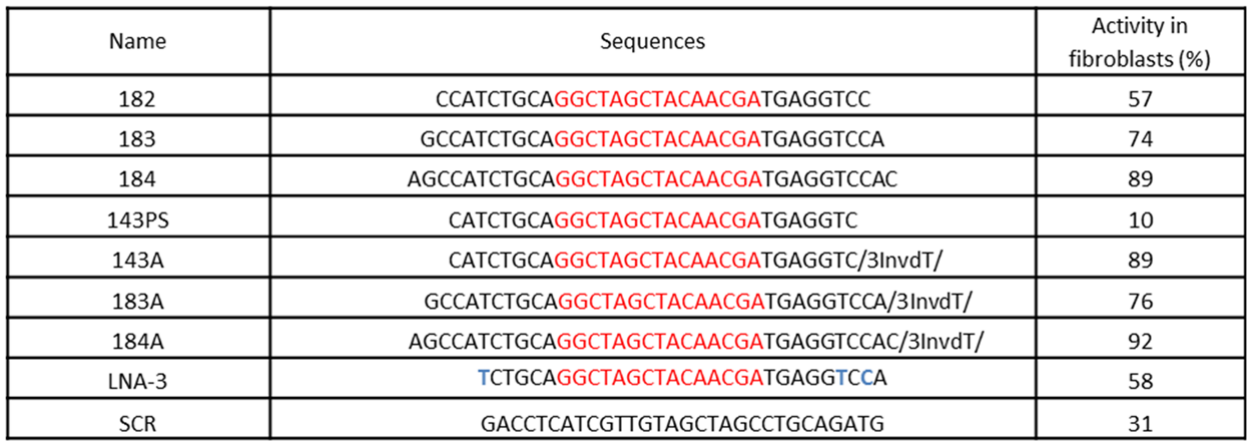
Second-generation DNAzymes derived from RNV143, targeting the *ITGA4* mRNA. The activity of DNAzymes directly correlated with the percentage of *ITGA4* mRNA knockdown. Activities of DNAzymes were calculated as described in Materials and Methods. The catalytic motifs are shown in red, the arm regions are in black and the residues modified with LNA are in blue. The sequences are from 5′ → 3′.

Based on the initial screen, the best performing DNAzyme RNV143 was selected for further modifications. Many studies have shown that increasing the hybridisation arms on either side of the catalytic motif can increase the binding affinity and efficacy^16–18^. In our study, the first generation of DNAzymes initially had 8 nucleotides on one arm and 7 on the other. Several studies showed that the optimal arm lengths vary from 7 to 10 nucleotides long^16–21^. Therefore, the length of RNV143 was systematically increased at the end of both arms, and then the efficacy of the modified DNAzymes was verified. One, two and three nucleotides were added to both arms of RNV143 and named RNV182, RNV183 and RNV184 respectively (Fig. 3). The catalytic activities of these second generation DNAzyme candidates were analysed in human primary fibroblasts as described above. The transfections were repeated at least twice. Notably, a decrease in activities were observed for RNV182 (57%) and RNV183 (74%) at 600 nM compared to the parent DNAzyme RNV143 (84%) (Fig. 3). RNV184 with additional six nucleotides (three on each ends) was the only candidate that showed similar activity to the parent RNV143, with 89% knockdown of *ITGA4* mRNA. These results showed that increasing the arm length did not dramatically improve the efficacy of DNAzyme RNV143 in fibroblasts.

Next, we explored the improvement of nuclease stability of the DNAzymes since the natural nucleotide monomers are rapidly degraded *in vivo*. First, we introduced phosphorothioate (PS) linkages to the arm regions of RNV143 to improve nuclease resistance and named RNV143PS. However, RNV143PS failed to cleave *ITGA4* RNA when transfected into the human fibroblasts. Only 10% of the *ITGA4* transcript was degraded when treated with RNV143PS while nearly 29% of the *ITGA4* transcript was non-specifically knocked down by the scrambled control (Fig. 3). Then we introduced another chemical modification, an ‘inverted dT’ (Fig. 1B) at the 3′ end of RNV143, RNV183 and RNV184, the DNAzymes with high activities, and the new generation DNAzymes were named RNV143A, RNV183A and RNV184A respectively. RNV143A, RNV183A and RNV184A slightly improved the activities of DNAzymes (89%, 76% and 92% respectively; Fig. 3).

We also investigated the potential use of locked nucleic acid (LNA) nucleotides in DNAzymes. Two LNA nucleotides were incorporated to both arms of RNV183 at positions 2, 5 and 31, 35. However, RNV183 with 4 LNA modifications was not effective (did not cleave *ITGA4*) when tested in cells (data not shown). We believe that this may be due to the formation of secondary structures caused by LNAs within the arms of the DNAzymes that could potentially affect the catalytic motif (sequence of the LNA modified RNV183 and the mfold^22^ predicted structures are shown in Supplementary Material, Figure S1). To limit this secondary structure formation, we truncated the LNA modified RNV183 by four nucleotides from the 5′ end to generate RNVLNA-3 (Fig. 3) with one LNA nucleotide on the truncated arm and two LNA nucleotides on the other non-truncated arm. This truncation is predicted to limit the secondary structure formation, and promote its ability to bind to the *ITGA4* mRNA. Transfection with RNVLNA-3 showed 58% knockdown of *ITGA4* transcript indicating that the truncation helped to improve the activity, although the efficacy of *ITGA4* knockdown was not efficient (Fig. 3).

### *In vitro* cleavage of *ITGA4* RNA template

To further verify the catalytic activity of DNAzyme towards the target region of *ITGA4* transcript, we performed the cleavage efficacy *in vitro* using a synthetic fluorescein dye (FAM)-labelled RNA target composed of exon 9 region of the *ITGA4* transcript. The experiments were performed by incubating DNAzymes with FAM-labelled RNA template in the presence of divalent metal ions and the products were separated and analysed on polyacrylamide gels. Briefly, 1.76 µM DNAzymes were incubated with 1.76 µM FAM-conjugated *ITGA4* RNA in the presence of Mg^2+^ divalent cations for 30 min, 60 min and 120 min at 37 °C. The reactions were stopped by adding 10 µL of formamide solution. The products were then separated on a 15% denaturing polyacrylamide gel and visualised using Fusion FX Vilber Lourmat imager. The cleaved products of the 34mer full length FAM-conjugated RNA was expected to be 18 nucleotides. A scrambled (SCR) sequence and RNV143 mutants with different mutations within the catalytic region of RNV143 were used as negative controls in parallel and an untreated (UT) sample with no DNAzyme was also included.

**Figure 4:**
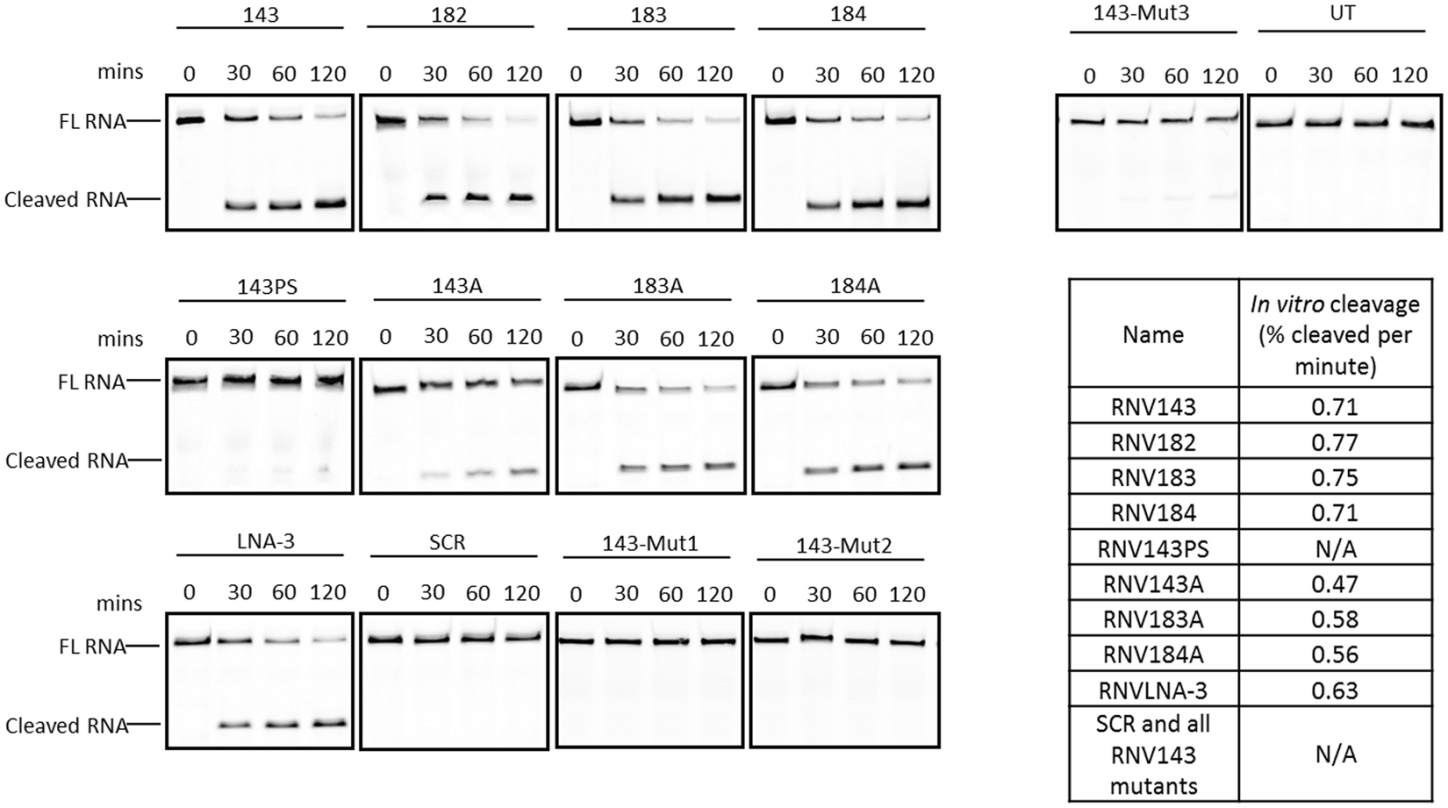
*In vitro* cleavage of the FAM-conjugated *ITGA4* RNA template composed of exon 9 region (34 nucleotides) by RNV143 and its derivatives. FL RNA, full length; FAM-conjugated RNA; cleaved RNA; the cleaved FAM-conjugated *ITGA4* RNA (18 nucleotides long). The FAM- conjugated template RNA is a small region of the *ITGA4* transcript complementary to the hybridisation arms of the DNAzymes of interest. The gel images were cropped for better overview. The original images are shown in Figure S3 (Supplementary Information). The table shows the cleavage rate in %/min which was calculated as described in Materials and Methods section.

Although variable efficiencies were observed for these DNAzymes in fibroblasts, RNV143, RNV182, RNV183 and RNV184 showed similar cleavage efficiency *in vitro* (around 0.7%/min) (Fig. 4). In general, the *in vitro* cleavage rates of the modified DNAzymes were slower than that of their parent oligonucleotides. Notably, RNV143PS showed very low cleavage efficiency with small percentage of cleaved FAM-conjugated RNA appearing after 120 minutes of incubation. Similar results were observed for RNV143-Mut3 while the other mutant DNAzymes and the SCR sequence showed no cleavage (Fig. 4).

### Nuclease stability analysis of DNAzymes

**Figure 5:**
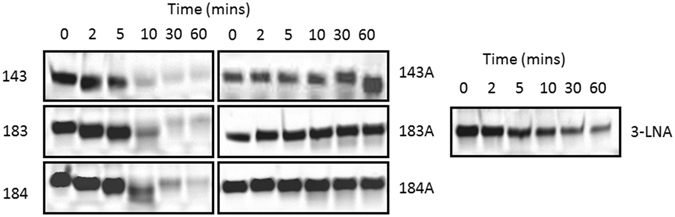
Phosphodiesterase degradation analysis of DNAzymes that showed high efficacy in the cleavage of *ITGA4* RNA *in vitro* and knockdown of *ITGA4* RNA in fibroblasts. The gel images were cropped for better overview. The original images are shown in Figure S4 (Supplementary Information).

High nuclease stability is paramount towards the clinical development of oligonucleotides. Towards this goal, we tested the stability of DNAzymes RNV143, RNV183, RNV184, RNVLNA-3, RNV143A, RNV183A, and RNV184A using snake venom phosphodiesterase, a harsh enzyme with very high 3′ → 5′ exonuclease activity. DNAzyme candidates were incubated with the enzyme at 37 °C and samples were collected at different time points (0, 30, 60 and 120 minutes) followed by the products analysis on 15% denaturing polyacrylamide gels. As expected the stability was increased by increasing the arm lengths of the DNAzymes and RNV184 (36 nt long) was found to be the most stable compared to RNV183 and RNV143 (Fig. 5). RNVLNA-3 with LNA modifications (known for high nuclease stability^23, 24^) was more stable than RNV143, RNV183 and RNV184. A weak product band was visible even after 60 minutes of incubation. Remarkably, the DNAzymes with inverted dT nucleotide at 3′-end (RNV143A, RNV183A and RNV184A) showed highest stability with no significant degradation even after 1 hour incubation with phosphodiesterase (Fig. 5).

## Discussion

We have successfully identified a DNAzyme that cleaves the *ITGA4* transcript both *in vitro* and in human primary fibroblasts by screening different DNAzyme constructs with 10–23 catalytic motif and 8–17 catalytic motif targeting different sites of *ITGA4* mRNA. The DNAzyme was not only capable of downregulating the *ITGA4* transcript *in vitro* and in human primary fibroblasts but also showed high resistance to exonuclease degradation. Our study thus provides a novel approach for downregulating *ITGA4* transcript that may have therapeutic potential towards the treatment of MS where ITGA4 is a validated target for tackling inflammation.

Cleavage by first generation DNAzymes with either 10–23 or 8–17 catalytic motif targeting five different cleavage sites of the *ITGA4* transcript showed variable cleavage efficiencies (Fig. 2B). The best cleavage efficiency was achieved with the 10–23 catalytic motif DNAzymes designed to cleave a site located in exon 9 of the *ITGA4* transcript. The variable target-cleaving efficiencies observed for different first generation DNAzymes targeting different regions of the *ITGA4* transcript could be due to the structure variations that might affect the accessibility of target sequences. This is consistent with previous observations by Vester *et al*.^17, 21, 25^. The cleavage efficiency of DNAzyme has been proposed to be dependent on the structural complexity of the RNA that is targeted^17, 26^. In a comparison between different substrate structures, Vester *et al*., targeted three different cleavage sites of either a short (17 nucleotides) unstructured RNAs; or longer (58 nucleotides) RNAs with stable secondary structures; or longer (2904 nucleotides) RNAs with both stable secondary and tertiary structures^17, 26^. The results indicated that only one cleavage site out of three could be efficiently cleaved by the DNAzymes and the cleavage of the short unstructured RNAs were more efficient than the longer structured RNA^17, 26^. This implies that the accessibility of the purine-pyrimidine sites differs in different regions of the *ITGA4* (1032 bp long) transcript due to the formation of different secondary and tertiary structures in the transcript inside the cells, leading to differences in the ability of DNAzymes to cleave them.

The optimal arm length may vary for different DNAzymes and there is no evidence on whether the arms should be asymmetrical or symmetrical for optimal catalytic activity. In addition, the GC content may also have an effect on the catalytic efficiency^16, 18, 21, 27^, but how it is affected is unclear^16, 18, 19^. In parallel, increasing the DNAzyme binding arm length would increase DNAzyme- target affinity as it enhances the heteroduplex stability^28^, but it could also result in slower product release that would inhibit or decrease the rate of multiple turnovers. Therefore, longer binding arms may not always produce higher catalytic efficiency^14, 29^. Using the best candidate RNV143 from the first screen, we further tested the impact of DNAzyme arm lengths aiming for increased efficiency. However, the catalytic efficiency was decreased in most DNAzymes with longer arms. We speculate that increasing the arm length of the DNAzyme RNV143 could increase the binding affinity of DNAzymes to the target RNA but result in slower product release and turnover^26^. For DNAzymes to have therapeutic potential, it is desirable to have high turnover.

Although we have identified a DNAzyme with high cleavage efficiency, one of the disadvantages of unmodified DNAzymes, similar to antisense oligonucleotides, is their vulnerability to nucleases *in vivo* and therefore, nuclease resistance is a critical characteristic of their physical property^30^. The suitable solution to increase nuclease resistance is to introduce chemical modifications^30^. Several modifications have been successfully introduced into the oligonucleotides including sugar modifications such as LNA^21, 22^, and inverted thymidine and backbone modification such as PS^30^. In line with this, we modified several of our efficient DNAzyme candidates, RNV143 and its two derivatives RNV183 and RNV184 with PS, LNA and inverted-dT. When modified with PS modification (RNV143PS), RNV143 resulted in decreased cleavage efficiency both *in vitro* (Fig. 4) and in human fibroblasts (Fig. 3). PS modification typically enhances the stability of the oligonucleotide against nucleases, however, they compromise the affinity to their target in some cases, which could result in decreased cleavage efficiency^30^. Similar results were also observed in another study that further support our observation that PS modification may not be ideal for developing efficient DNAzymes^19^. Although truncation and incorporation of LNA to the DNAzymes increased resistance to nuclease (Fig. 5), the activity was decreased (Fig. 3). The reduction in the activity may be due to a combination of reduced size and slower DNAzyme dissociation from the mRNA target based on the high affinity of LNA nucleotides^19^. Among all modifications tested, incorporation of inverted dT (RNV143A, RNV183A and RNV184A) was found to be the best chemistry in this case, since it not only conferred nuclease resistance (Fig. 5) but also achieved high activity (89%, 76% and 92% respectively) (Fig. 3). The result is also consistent with a previous study performed by *Schubert et al*.^19^. Additionally, we have also performed a human serum degradation experiment to observe the stability of the DNAzymes in human serum (Figure S4 in Supplementary Material). RNV143, RNV182, RNV183 and RNV184 showed similar stability (degradation observed after 2 hours incubation) in human serum and as expected their modified counterparts RNV143A, RNV183A and RNV184A showed better stability (~26–50% DNAzymes remained after 6 h incubation). Interestingly, RNVLNA-3 conferred the best stability in human serum, however as discussed above, its activity in cells was decreased (Fig. 3).

We observed a discrepancy between the *ITGA4* knock down observed in fibroblasts and the *in vitro* cleavage for the same DNAzymes. Although variable efficiencies are observed for second generation DNAzymes in human primary fibroblasts (Fig. 3), the *in vitro* data does not reflect this (Fig. 4). It is expected that *cell-free* conditions are incomparable to the crowded *cell-culture* environment and the mRNA folding and accessibility of the template may significantly influence the success of experiments within the cells. It is also expected that the *ITGA4* RNA expressed in fibroblasts (1032 base pairs long) is much longer and can adopt complex secondary and tertiary structures while the synthetic short single stranded RNA (34 nucleotides long) used for the *in vitro* cleavage assay may not form any secondary or tertiary structures and therefore it is much easier for the DNAzyme to access and cleave. However, the DNAzymes that knocked down *ITGA4* transcript in fibroblasts also showed the ability to cleave the synthetic *ITGA4* transcript template *in vitro* and from this we may suggest that the *ITGA4* knock down observed in the fibroblast could be due to the DNAzymes-mediated cleavage of *ITGA4* transcript. Furthermore, we designed three RNV 143 mutants RNV143-Mut1, RNV143-Mut2 and RNV143-Mut3 to investigate the possibility of antisense effect rather than DNAzyme cleavage. RNV143-Mut1 and RNV 143-Mut2 are double mutants designed based on the paper by Wang *et al*. that suggested that these double mutants were critical for activity in the hammerhead and the stem loop respectively^31^. Both double mutants may be important for catalytic activity as they abolished the *in vitro* cleavage activity (Fig. 4). However, RNV143-Mut3 showed very small cleavage activity indicating that the nucleotide mutated may not be crucial for catalytic activity, supporting the previous study by Wang *et al*.^31^. These controls also suggest that the gene silencing effects seen in cells by RNV143 may be due to DNAzyme cleavage rather than the antisense effect. The cleavage rates of the DNAzymes observed here is about half of that reported by Schubert *et al*.^19^. However, we performed the *in vitro* cleavage reactions with equal molar ratio of the DNAzyme to substrate in the presence of 0.5 mM MgCl_2_ while Schubert *et al*., performed the reactions with 10-fold excess of DNAzyme to substrate in 10 mM MgCl_2_^19^.

In conclusion, we identified DNAzymes that are capable of cleaving the *ITGA4* transcript in human primary fibroblasts. The DNAzyme candidate RNV143 targeting exon 9 of the *ITGA4* transcript showed 92% knock down of the *ITGA4* transcript and the catalytic ability of the DNAzyme was verified by *in vitro* cleavage assay. Furthermore, introducing a chemical modification such as an inverted dT at the 3′ end (RNV143A) significantly improved the stability while maintaining efficient catalytic activity. Although RNV143A needs further validations, based our current results, we firmly believe that the candidate could provide therapeutic benefits.

## Methods

### DNAzyme design and synthesis

DNAzymes with either stem loop or hammer head conformation were designed for the selected exons (Fig. 2B) and the oligonucleotides were ordered from IDT. The LNA modified DNAzyme was made in-house by ABI Expedite 8909 nucleic acid synthesis system in 1 µM scale, deprotected by NH_4_OH at 55 °C overnight and purified by NAP column (GE Health care).

### Cell propagation and transfection

Cell cultures media and supplements were purchased from (Life technologies, Australia) unless specified. Normal human fibroblasts were propagated in Dulbecco’s modified Eagle’s medium supplemented with GlutaMAX™ and 10% fetal bovine serum. Transfections were performed in 24 well-plate format with approximately 15,000 cells/well. The cells were seeded one day before transfecting with the DNAzymes complexed with Lipofectamine 3000® transfection reagent (Life Technologies, Australia) per the manufacturer’s protocol. Transfection was carried out for 24 hours before harvesting RNA for transcript analysis. RNA was extracted using Direct-zol RNA MiniPrep Kit (Zymo Research, USA) following manufacturer’s protocol.

### RT-PCR assays

50 ng of total RNA was analysed using a Superscript III One-Step RT-PCR System (Life technologies, Australia) and reaction conditions are as follows: 55 °C for 30 min, 94 °C for 2 min, 28 rounds of 94 °C for 30 sec, 55 °C for 30 sec and 68 °C for 1 min 30 sec. Exon 1–10 was amplified using primer pair 1F (5′-gagagcgcgctgctttaccagg-3′) and 10R (5′-gccatcattgtcaatgtcgcca-3′); exon 9–20 using primer pair 9F (5′-ggatcgtactttggagcttctg-3′) and 20R (5′-gcatgcactgtgatactgaggt-3′). The products were analysed on 2% agarose gels.

### Image analysis of the gel from gel electrophoresis

Densitometry (measuring the band intensity) of the bands was performed using Image J Software^32^. The band intensity of the *ITGA4* bands in different DNAzymes treated samples were measured and normalised to the band intensity of the corresponding *CYCD* bands before comparing to the band intensity of the untreated samples. The percentage of *ITGA4* transcript knockdown by DNAzymes in fibroblasts was expressed as activity of DNAzyme.

### *In vitro* cleavage assay

4.4 µL of 20 µM DNAzyme was incubated with equal molar concentration of FAM-conjugated *ITGA4* RNA (5′-FAM-CUGUGCUGUGGACCUCAAUGCAGAUGGCUUCUCA-3′) in 5 µL of buffer containing Mg^2+^ divalent cations (10 mM MgCl_2_) at 37 °C. The reaction was stopped by adding 10 µL of formamide to 10 µL of the reaction mixture at 0, 30 mins, 60 mins and 2 hours. Scrambled DNAzyme RNV174, RNV143-Mut1 (5′-CATCTGCAGGCTA**AA**TACAACGATGAG-3′), RNV143-Mut2 (5′-CATCTGCA**AA**CTAGCTACAACGATGAG-3′) and RNV143-Mut3 (5′-CATCTGCAGGCTAGC**A**ACAACGATGAG-3′) were used as negative controls and the untreated samples did not have any DNAzyme. The mutated bases are in bold and the two double mutants RNV143-Mut1 and RNV 143-Mut2 were designed based on a previous paper^31^ The reaction mixtures were separated on a 15% polyacrylamide gel/7 M urea for 50 mins at 13 W. The gel was visualised using the Fusion FX Vilber Lourmat Imager (Fisher Biotech).

### Image Analysis of the gel from *in vitro* cleavage assay

Densitometry (measuring the band intensity) of the bands was performed using Image J Software^32^. The band intensity of the full length RNA bands for different time points were measured and normalised to the combined band intensity of both cleaved and full length RNA bands for different time points and plotted on excel as the % of uncleaved RNA. The same analysis was repeated to calculate % of cleaved RNA. The slope of the curve was used to calculate the % cleaved per minute.

### Phosphodiesterase assay

5 µM DNAzyme was incubated with 0.00001 U phosphodiesterase from *Crotalus adamanteurs* venom (Sigma Aldrich) at 37 °C. At different time points 0, 2 mins, 5 mins, 10 mins, 30 mins and 1 hour, 10 µL of formamide was added to equal volume of the reaction mixture to stop the reaction. The reaction mixture was separated on a 20% polyacrylamide/7 M urea gel. The gel was stained with ethidium bromide for 10 minutes and destained in water for 10 minutes before visualizing under UV light using Bio-Rad Molecular Imager ChemiDoc XRS Imaging System.

### Human serum degradation assay

5 µM DNAzyme was incubated in human serum at 37 °C. At different time points 0, 30 mins, 60 mins, 2 hours, 4 hours and 6 hours, 10 µL of formamide was added to equal volume of the reaction mixture to stop the reaction. The reaction mixture was separated on a 15% polyacrylamide/7 M urea gel. The gel was stained with ethidium bromide for 10 minutes and destained in water for 10 minutes before visualizing under UV light using Bio-Rad Molecular Imager ChemiDoc XRS Imaging System. The images are shown in Figure S4 (Supplementary Information).

## Electronic supplementary material

Supplementary Information
