# Supplementary material for: Novel Chemically-modified DNAzyme targeting Integrin alpha-4 RNA transcript as a potential molecule to reduce inflammation in multiple sclerosis
Source: Sci Rep. 2017 May 9;7:1613. doi: 10.1038/s41598-017-01559-w (PMC5431638; doi:10.1038/s41598-017-01559-w)
Supplement: Supplementary file 1 — Supplementary Information [file 41598_2017_1559_MOESM1_ESM.pdf]

Supplementary Information

Novel Chemically-modified DNAzyme targeting Integrin alpha-4 RNA transcript as potential molecule to reduce inflammation in multiple sclerosis

Madhuri Chakravarthy [1,2], May T. Aung-Htut [1], Bao T. Le [1,2,], and Rakesh N. Veedu [1,2*]

[1]Centre for Comparative Genomics, Discovery Way, Murdoch University, Perth, Australia-6150

[2] Western Australian Neuroscience Research Institute, Murdoch, Perth, Australia- 6150

[*]Address for Correspondence: R.Veedu@murdoch.edu.au

mfold structures

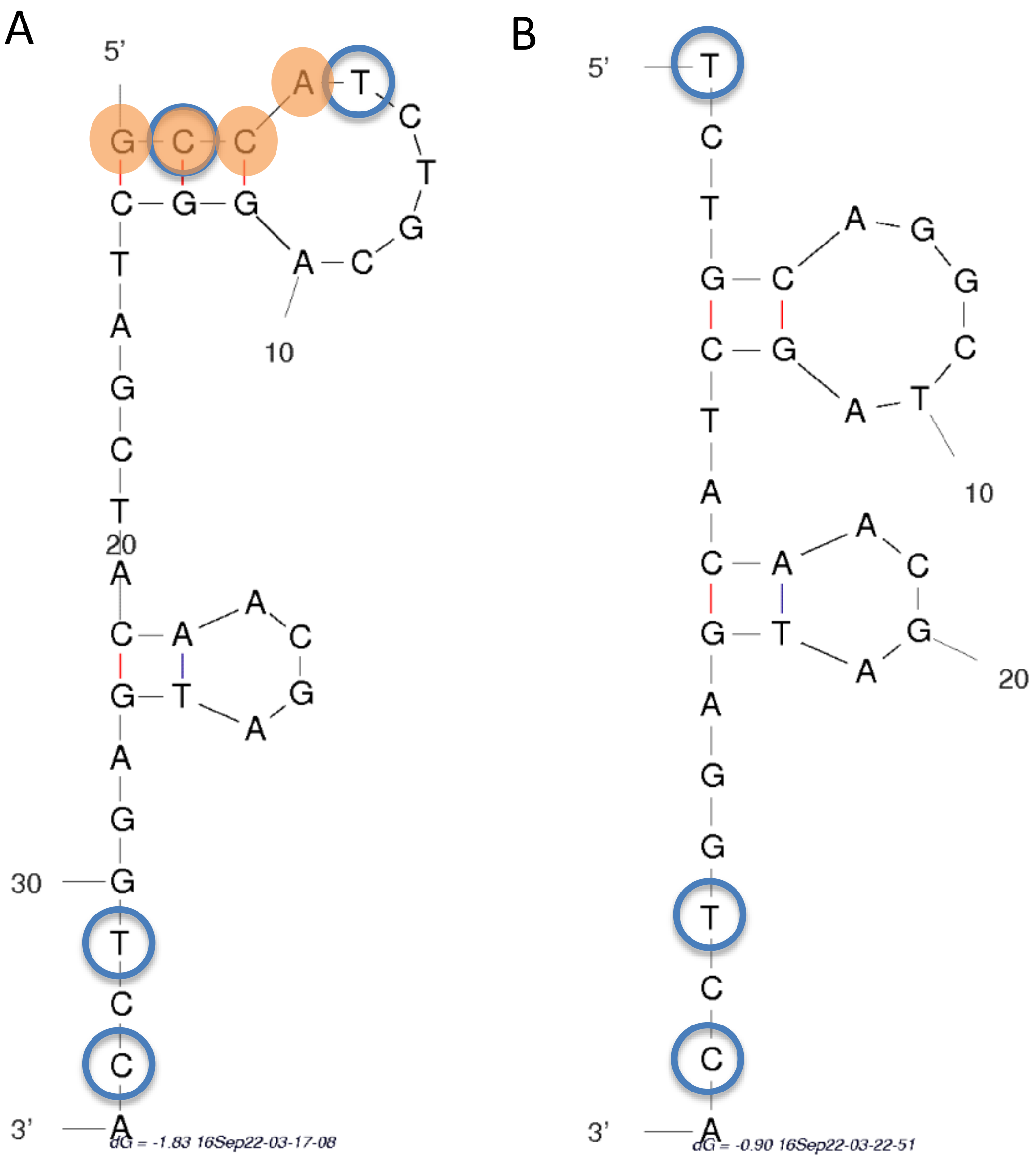

Supplementary Figure S1. The structure of the DNAzyme RNV183 (A) and LNA-3 predicted by mfold (B).[1] 183 was modified to develop LNA-3 by truncation of four residues at the 5’ end (highlighted in orange) and modification of bases as LNA (blue circles).

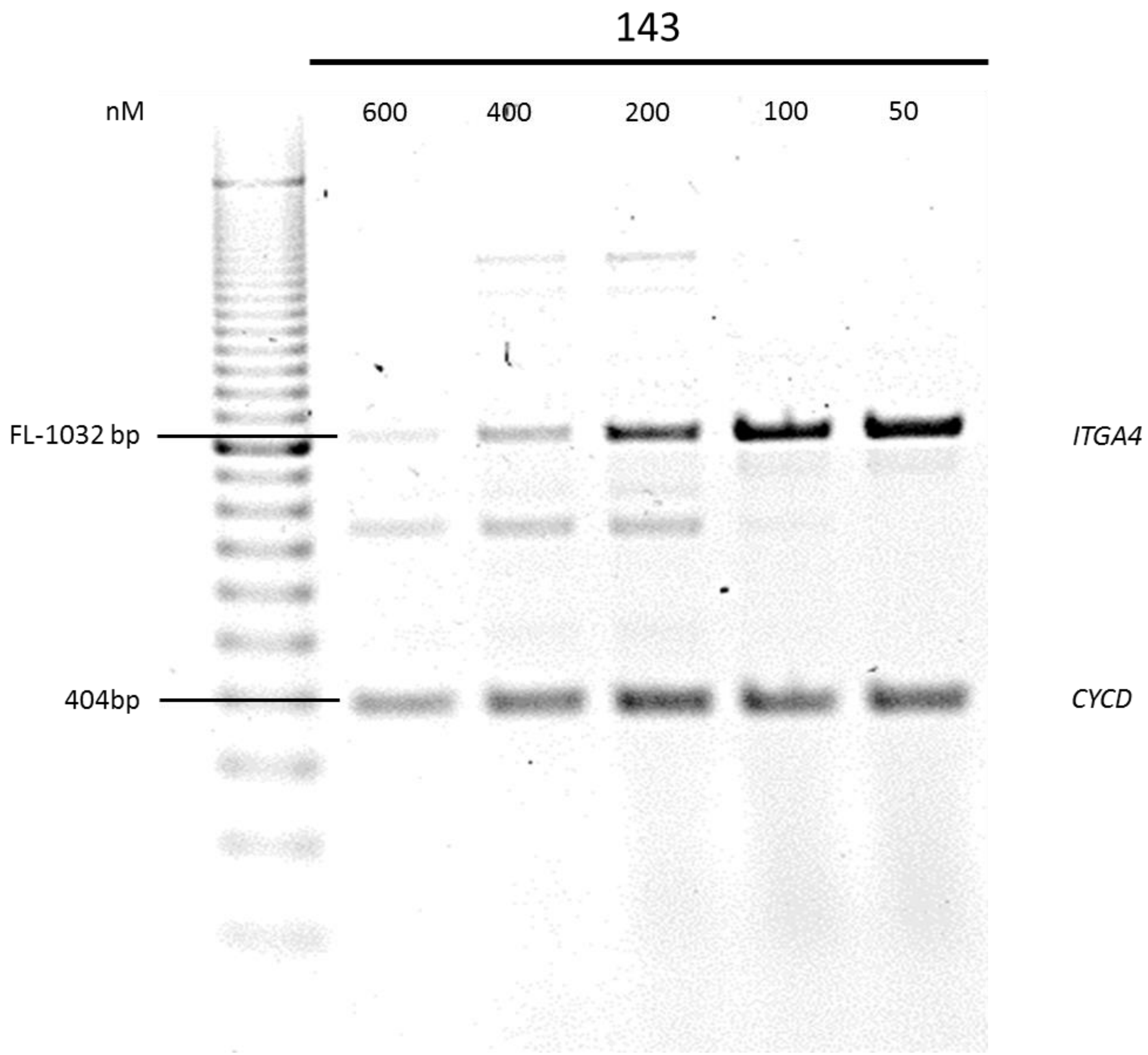

Supplementary Figure S2. The RT-PCR products after treatment with RNV143 is shown here. FL, full-length; CYCD was used as a loading control. [The gel in this figure is the original gel representing the gel in Figure 2 of the article. The cropped gel has been shown in Figure 2 of the article due to non-specific bands that exist and other unimportant samples that exist between the desired samples.]

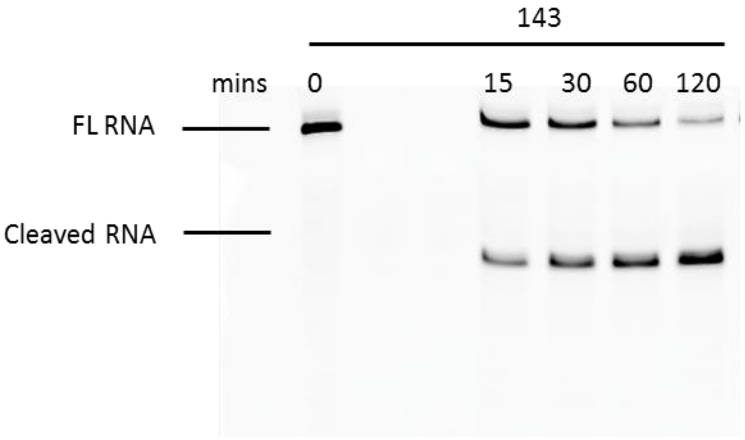
143
mins 0 15 30 60 120
FL RNA
Cleaved RNA

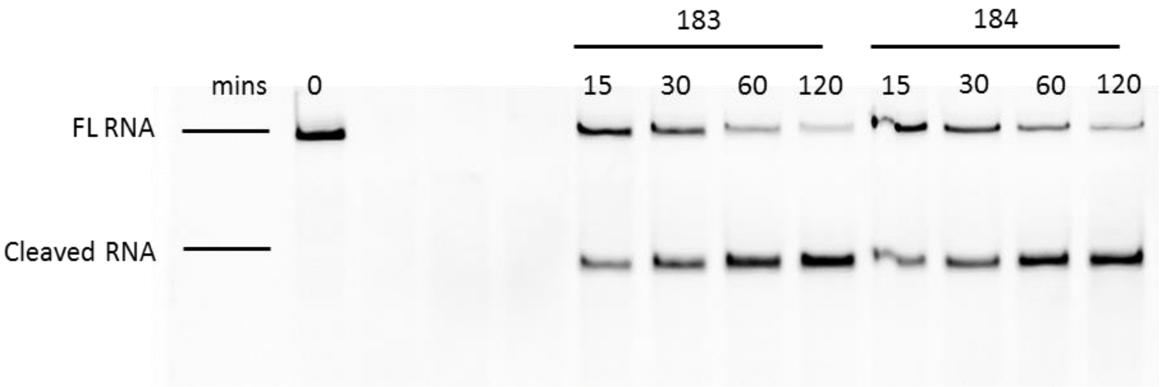
183
184
mins 0 15 30 60 120 15 30 60 120
FL RNA
Cleaved RNA

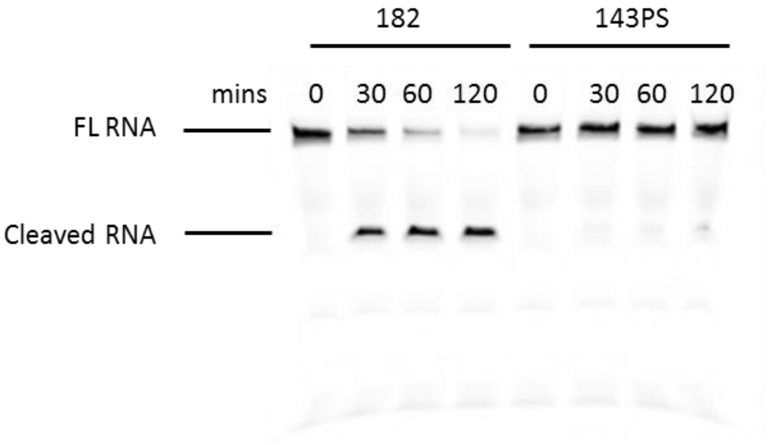
182
143PS
mins 0 30 60 120 0 30 60 120
FL RNA
Cleaved RNA

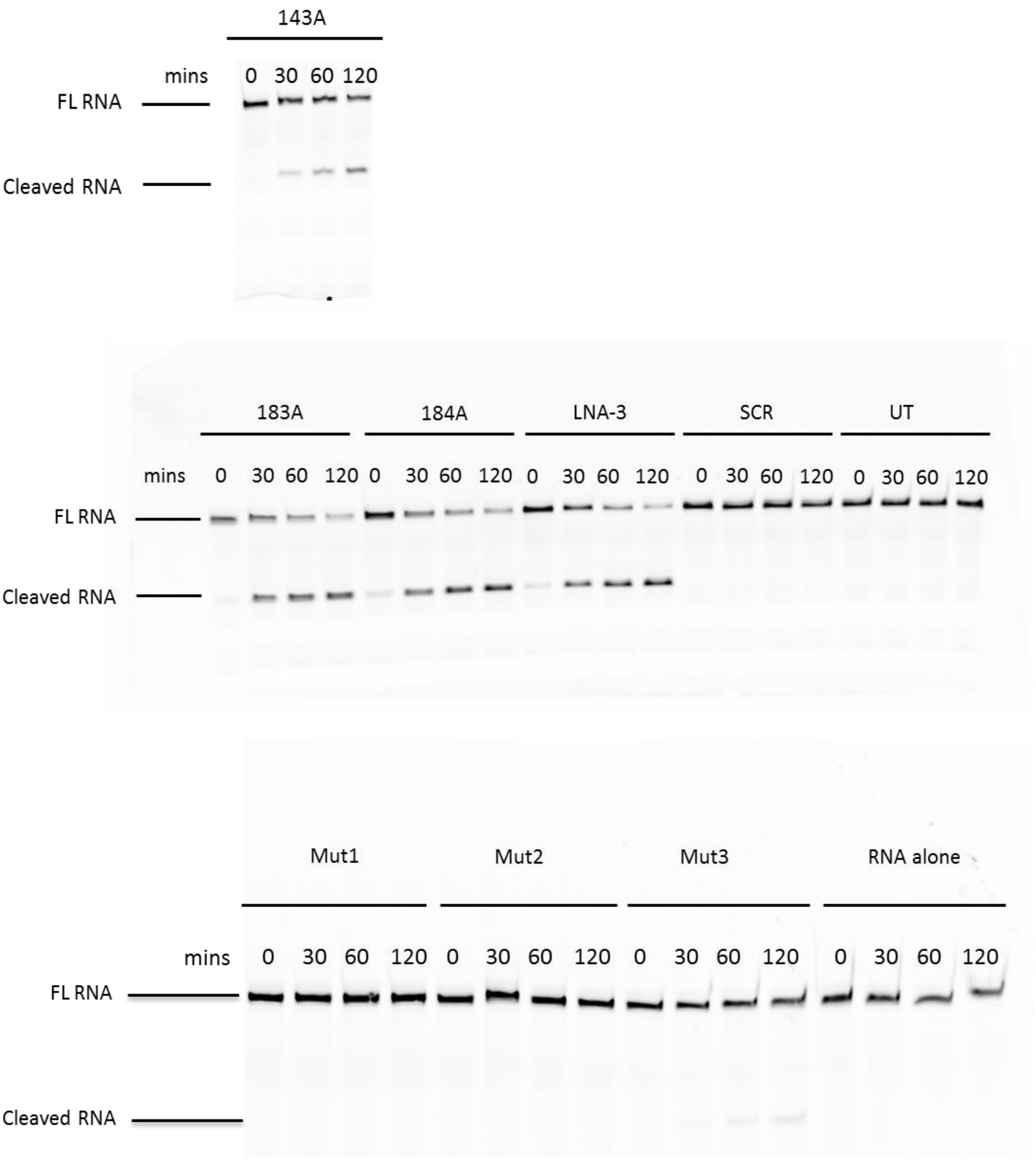

Supplementary Figure S3. *In vitro* cleavage of the FAM-conjugated *ITGA4* RNA template composed of exon 9 region (34 nucleotides) by RNV143 and its derivatives. FL RNA, full-length FAM-conjugated RNA; cleaved RNA, the cleaved FAM-conjugated *ITGA4* RNA (18 nucleotides long). The FAM-conjugated template RNA is a small region of the *ITGA4* transcript complementary to the hybridization arms of the

DNAzymes of interest. [The gels in this figure are the original gels representing the gels in Figure 3 of the article. For DNAzymes 143, 183 and 184 there are 5 time points (0, 15, 30, 60 and 120 mins) in the original gels but these were cropped to include only 4 time points (0, 30, 60 and 120 mins with timepoint 15 mins excluded) in the article to ensure consistency with the data for the other DNAzymes. The order of the gels here are not the same as that represented in Figure 3 of the article and different experiments were run on different gel and therefore the data for each DNAzyme was cropped and arranged in the order seen in Figure 3 of the article.]

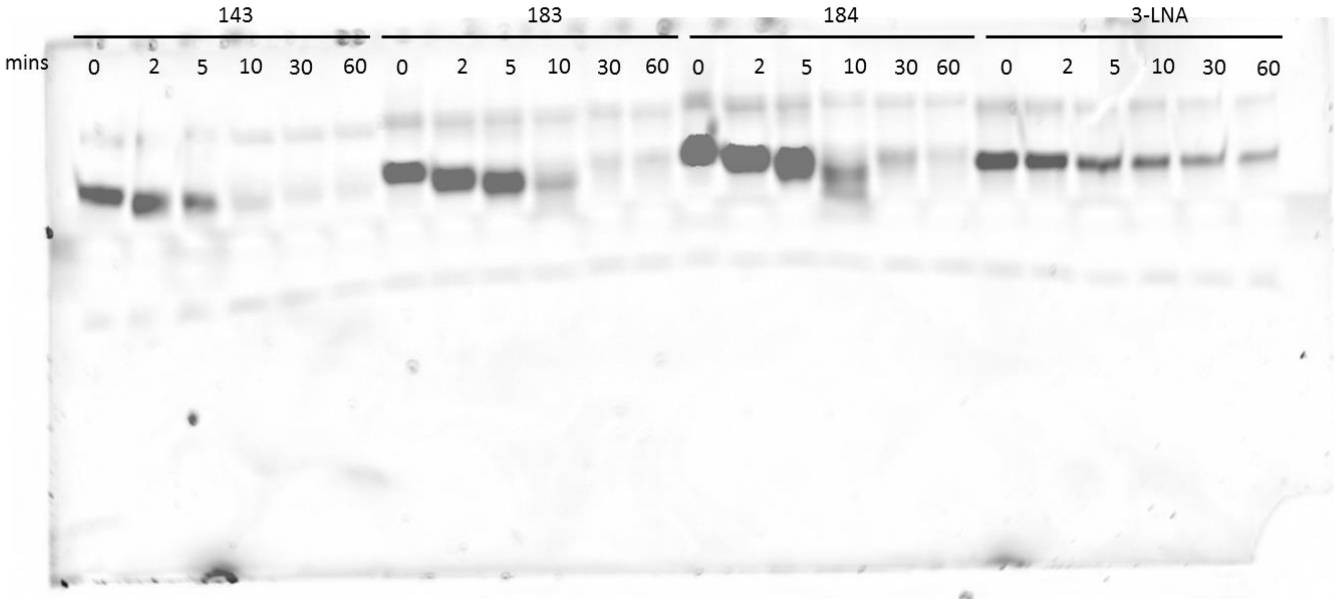

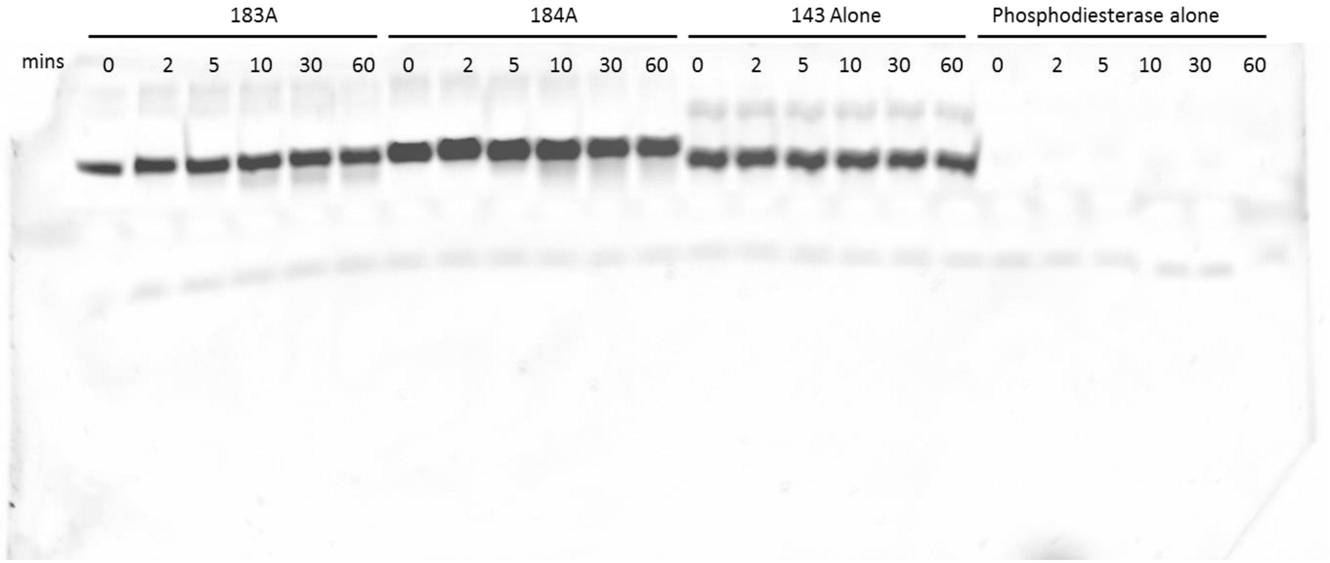

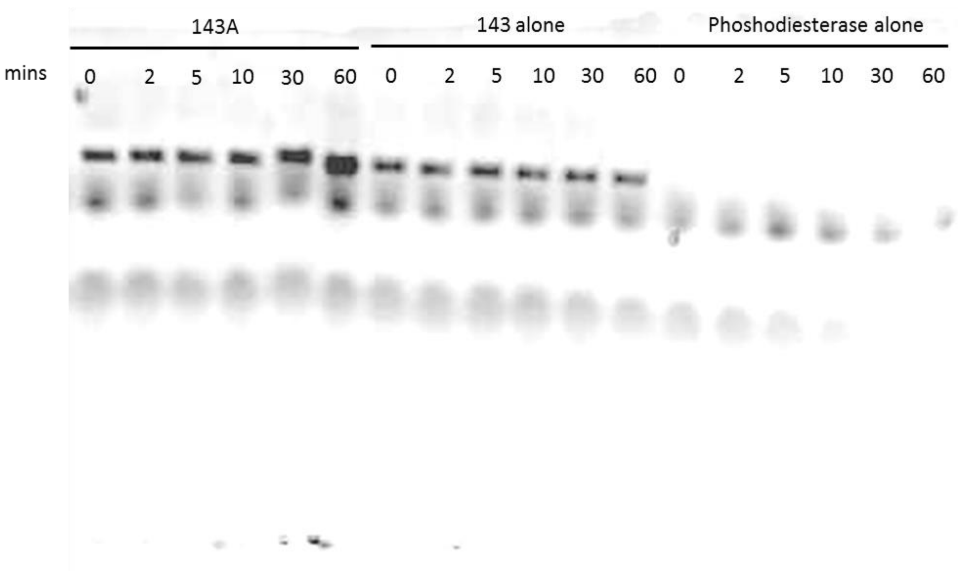

Supplementary Figure S4. Phosphodiesterase degradation analysis of DNAzymes that showed high efficiency in the cleavage of *ITGA4* RNA *in vitro* and knockdown of *ITGA4* RNA in fibroblasts. [The gels in this figure are the original gels representing the gels in Figure 4 of the article. The order of the gels here are not the same as that represented in Figure 3 of the article and different experiments were run on different gels and therefore the data for each DNAzyme was cropped and arranged in the order seen in Figure 4 of the article.]

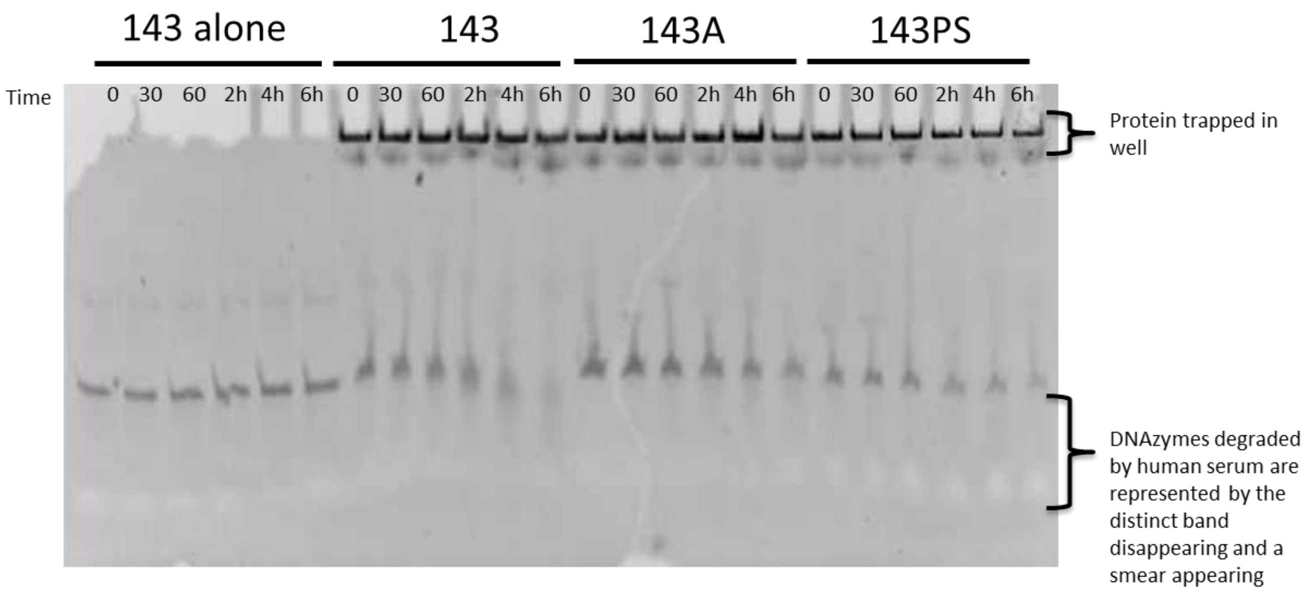
143 alone
143
143A
143PS
Time
0 30 60 2h 4h 6h 0 30 60 2h 4h 6h 0 30 60 2h 4h 6h 0 30 60 2h 4h 6h
Protein trapped in well
DNAzymes degraded by human serum are represented by the distinct band disappearing and a smear appearing

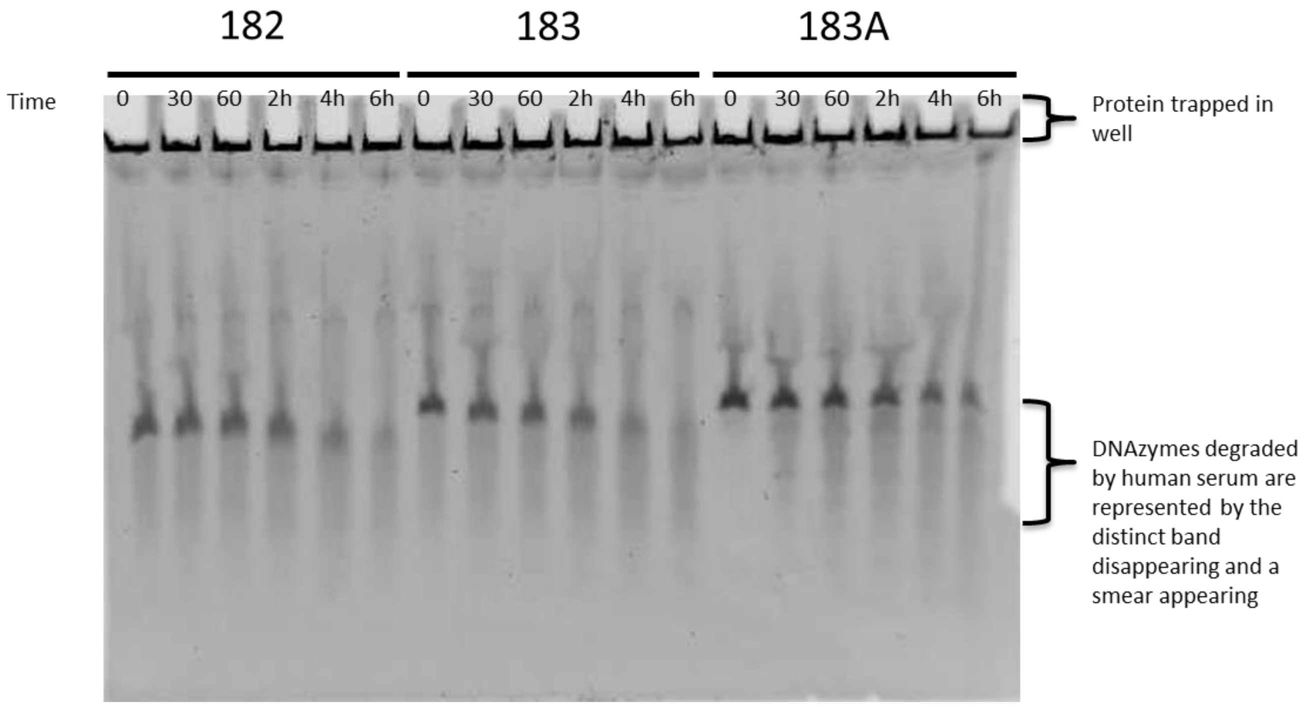
182
183
183A
Time
0 30 60 2h 4h 6h 0 30 60 2h 4h 6h 0 30 60 2h 4h 6h
Protein trapped in well
DNAzymes degraded by human serum are represented by the distinct band disappearing and a smear appearing

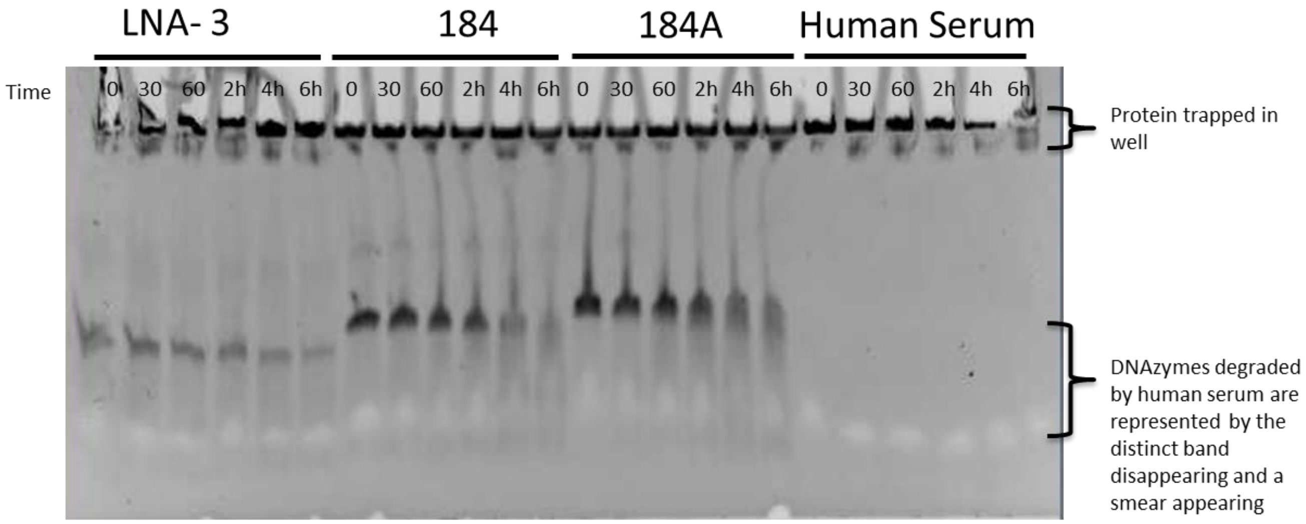

Supplementary Figure S5. Human serum degradation analysis of DNAzymes that showed high efficiency in the cleavage of *ITGA4* RNA *in vitro* and knockdown of *ITGA4* RNA in fibroblasts.

References:

1. M. Zuker, *Nucleic Acids Res*, 2003, **31**, 3406-3415.
